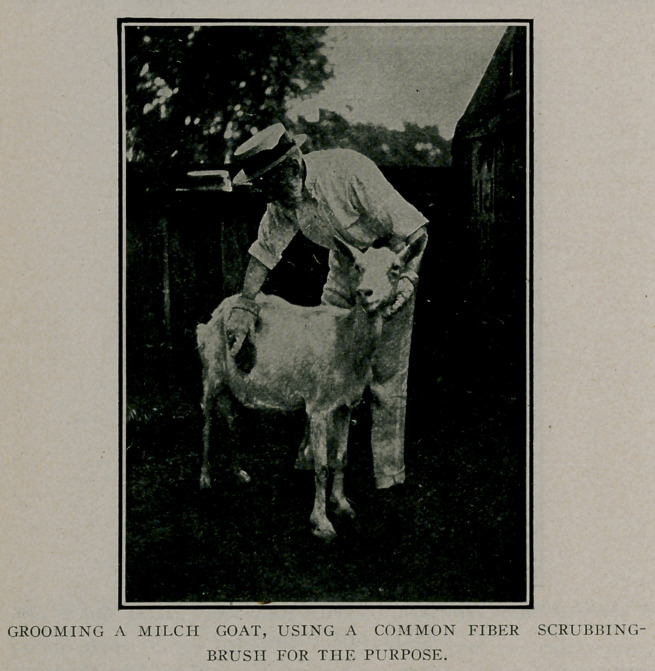# One Family’s Solution of the Milk Problem

**Published:** 1909-12

**Authors:** W. Sheldon Bull

**Affiliations:** Buffalo, N.Y.; Member of the British Goat Society


### BUFFALO MEDICAL JOURNAL.

VOL. LXV. DECEMBER, 1909. NO. 5

### ORIGINAL COMMUNICATIONS.

### One Family's Solution of the Milk Problem

#### A Satisfactory Experiment with Milch Goats

BY W. SHELDON BULL, Buffalo, N. Y.
Member of the British Goat Society.

[*From "Suburban Life" Magazine*, October, 1909.]

AFTER having devoted much thought to our family milk problem, it finally occurred to us that the only way to become independent of the milkman and his blue milk would be to install upon our premises a small dairy for the production of "home-grown" milk. Living as we did, however, in the residential section of Buffalo, a cow was out of the question, and our thoughts turned to milch goats, an acquaintance having recently imported from Switzerland several Saanen and Toggenburg goats. The fact that the milch goat is practically immune to tuberculosis predisposed us in her favor, and we decided to establish a small goat dairy in our back yard.

All our friends assured us that our experiment was doomed to failure on account of the goatish odor and flavor which the milk would be sure to have. These forebodings were without good reason, we have found, for clean goat's milk is odorless and as pleasant to the taste as the very best clean cow's milk, and it is richer and more digestible. We secured all the literature on the subject of milch goats that we could find, including several valuable publications issued by the United States Department of Agriculture. We learned that the all-important fact in the production of clean, healthful milk was to consider absolute cleanliness, in the animal, in the person who cares for her, and in the utensils which are used. Our environment is such, however, as to preclude all thought of our utilising the services of the good old Bossie Cow, as we live in the heart of the residence district of Buffalo with a Health Commissioner, in Dr. Ernest Wende, who is noted for the faithful and fearless performance of his duties in guarding the public health, and who enforces a strict compliance with the laws and ordinances relating to sanitation and hygiene, utterly regardless of one's "pull."

When it came to the question of actually obtaining the milch goat, we were at a loss where to turn. First we sought the foreign quarter of the city, and found a good goat which yielded two quarts of milk a day, and bought the animal. At our first attempt at milking, however, we discovered that her teats were so small that she could be milked only by the process known as "stripping," which is tedious and tiresome, and practically impossible for us with our untrained hands and muscles.

BABETTE, A MILCH GOAT, WAITING FOR THE DOOR OF THE MILKING-SHED TO BE OPENED.

Shortly afterward we took advantage of an opportunity offered us, and bought two fine, large, imported Saanen does, which had been in this country a sufficient length of time to have become thoroughly acclimated. These goats are hornless, have short white or cream-colored hair, and are much larger and have a more deer-like form and carriage than the common or garden variety. Like Jersey cows, they are somewhat lean and bony in appearance and, as a rule, fine milk-producers.

We found that the best time to buy common goats is usually in the fall of the year, when the pasturage becomes scarce. Then the goat owners are generally willing to part with even their best goats at very reasonable prices, rather than feed and house them through the long winter, when provender is high and stabling facilities inadequate.

Good common goats usually cost from five to fifteen dollars. As milch-goat breeding is not yet an established industry in this country, prices vary, and the quest for a good milker must be persevered in until satisfactory animals are secured. Should the would-be goat fancier prefer to invest in imported stock, however, the names and addresses of the few importers of the Saanen, and Toggenburg Swiss milch goats may be obtained on application to the Bureau of Animal Industry, United States Department of Agriculture, Washington.

Before purchasing our first goat, we fenced off the rear portion of our back yard for a goat paddock. In the far corner of this enclosure we built, out of some second-hand lumber we had on the place, a shed in which to stable our prospective purchases. Our reading had taught us that goats, in order to be kept in good health, must be kept dry and out of drafts, and yet given plenty of fresh air: so we took especial pains to make the shed wind- and water-tight, at the same time providing for its being thoroughly ventilated, making use of the "muslin system" of ventilation.

In addition to two half-bred kids presented us by our first nanny, a buck and a doe, this little stable afforded adequate shelter for our two large Saanen does, Babette and Nannette, and later also for their three thorough-bred kids, which, to our disappointment, were all "Billy goats."

At first, following the ordinary dairy practice, we milked in the stable. We soon became convinced, however, that we could greatly improve upon this method from a sanitary point of view, and at the same time add largely to our own personal comfort and convenience; for we found crouching down beside the goats, while milking them, very tiresome. So, after considerable planning and contriving, we converted three upright piano boxes into a milking shed. Here, sitting beside the milking platform, we milk at our ease, and under the most ideal sanitary conditions.

This little structure, situated at the other end of the paddock from the stable, has a door at each end, which, when both are opened, admit the fresh air and sunshine to every corner. It is at all times sweet and wholesome, free from dust and flies, and from animal odors and exhalations, as the goats are in it but twice a day, and then only one at a time while being milked.

By using a plentiful supply of baled shavings for bedding, and by cleaning up both night and morning, the stable is easily kept in a sanitary condition, as shavings are a good absorbent.

If there is no vegetable or flower garden convenient for which the goat manure can be used, it can be disposed of through one of the concerns, found in any large city, which make a business of carting away and selling manure.

We feed our goats three times a day in winter, when not being milked, and four times a day the rest of the year,—the last meal being given about 9 P. M. Their chief article of diet is hay, clover preferred, supplemented by bran and oats. We also give them clean kitchen leavings, such as stale bread, apple and

ON THE MILKING-PLATFORM, FEEDING AND READY TO BE MILKED.

potato skins (either baked or raw), carrot, beet and celery tops, pea-pods, and the like. We give them only as much hay or grain as they will eat at one feeding, which amount we soon learned to know by experience. The hay is placed in little racks above each small manger, the slats being sufficiently close together to prevent the goats from pulling down more than a mouthful at a time. In order to keep goats economically, this is important, as these fastidious and wasteful little animals will not eat anything once trodden under foot or soiled in any other way. In addition, we furnish them with brush-wood and tree trimmings, when obtainable, in order to indulge them in their deer-like propensity for browsing and bark-peeling.

It is most essential to the health and well-being of goats which are so closely confined as ours that they be thoroughly and frequently groomed. We brush the does, when in milk, twice a day in the open air just before milking. We use for this purpase, as shown in our illustration, small fiber scrubbing brushes costing five cents apiece. By using a pair of coarse and then a pair of fine brushes, we are able to keep both hair and skin of the animals perfectly clean and free from all manner of parasites. The canvas gloves, one of which appears so prom-

A SAANEN GOAT BROWSING ON THE HILLSIDE. THESE GOATS ARE EXCELLENT FORAGERS.

inently in the foreground of the illustration, cost ten cents a pair.

Although the price we paid for our imported Saanen—between forty and forty-five dollars apiece laid down in our backyard dairy—seemed high to us at the time we bought them, and several of our hereinbefore-mentioned friends have quite casually called our attention to the fact that we might have bought a good cow for less money, still we have felt no regret at having purchased them, as they have in the two years since then much more than paid for themselves and their keep.

What milk we do not need for our own household use we find no difficulty in selling as a nutrient for infants and invalids, as prescribed by their respective physicians. Sold for this purpose, it does not enter into competition with cow's milk at all, and readily brings twenty cents per pint.

In addition to the fact that our experiment has been more than self-supporting, we have been directly benefited in other ways which cannot be so accurately estimated in dollars and

GROOMING A MILCH GOAT, USING A COMMON FIBER SCRUBBING-BRUSH FOR THE PURPOSE.

cents, not the least among these good results being a decided improvement in health brought about in caring for the goats night and morning, an incentive to out-of-door exercise which proved a veritable boon to one engaged in sedentary office work. And then, too, we have enjoyed the luxury of being able to supply our own household with its daily supply of milk, which even our former carping friends are now willing to admit is absolutely free from any so-called goatish taste or odor, and as pure and fresh as every known precaution can make it. But, best of all has been the satisfaction we have felt each year in being instrumental, by means of our goats' milk, in tiding over the trying hot spell for several artificially fed babies in our neighborhood. Some of these little ones, ranging from three to fourteen months in age, had hardly more than a fighting chance for life, when, as a last resort, our goats' milk was prescribed for them by one of our leading baby specialists.

204 Ashland Avenue.

## Figures and Tables

**Figure f1:**
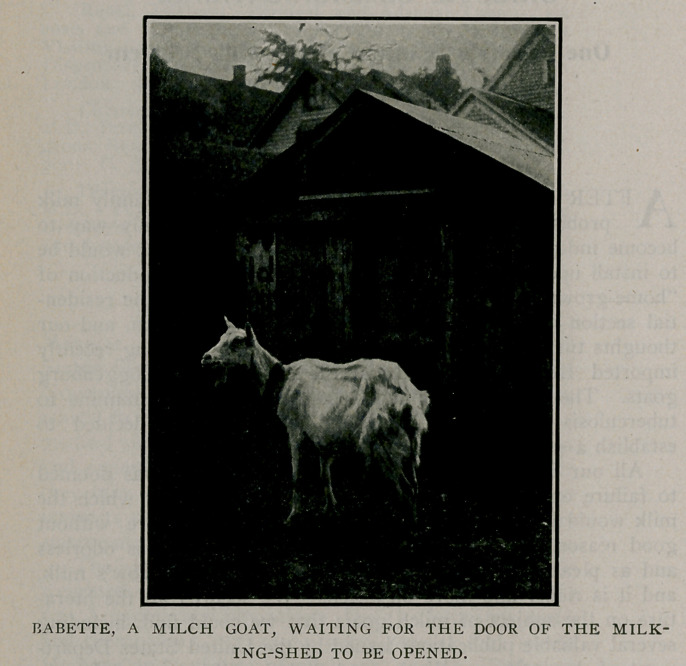


**Figure f2:**
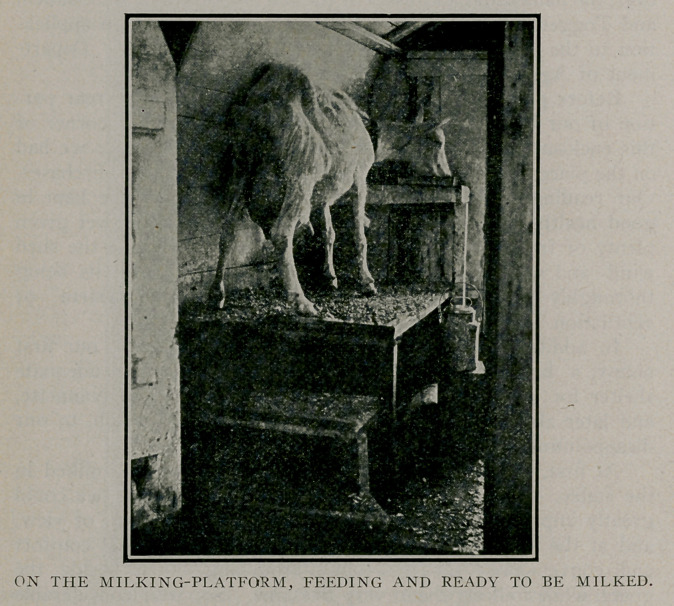


**Figure f3:**
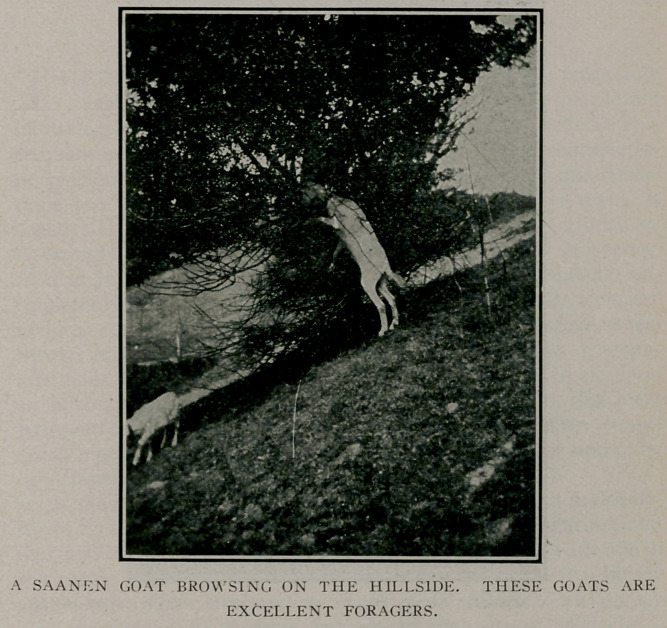


**Figure f4:**